# European Society of Cardiology Guideline-Adherent Antithrombotic Treatment and Risk of Mortality in Asian Patients with Atrial Fibrillation

**DOI:** 10.1038/srep30734

**Published:** 2016-08-08

**Authors:** Cheng-Hung Li, Chia-Jen Liu, Annie Y. Chou, Tze-Fan Chao, Ta-Chuan Tuan, Su-Jung Chen, Kang-Ling Wang, Yenn-Jiang Lin, Shih-Lin Chang, Li-Wei Lo, Yu-Feng Hu, Fa-Po Chung, Jo-Nan Liao, Tzeng-Ji Chen, Tsu-Juey Wu, Shih-Ann Chen

**Affiliations:** 1 Division of Electrophysiology, Cardiovascular Center, Taichung Veterans General Hospital, Taipei, Taiwan; 2 Institute of Clinical Medicine, and Cardiovascular Research Center, National Yang-Ming University, Taipei, Taiwan; 3 Division of Hematology and Oncology, Department of Medicine, Taipei Veterans General Hospital, Taipei, Taiwan; 4 Institute of Public Health and School of Medicine, National Yang-Ming University, Taipei, Taiwan; 5 Division of Cardiology, Department of Medicine, Lions Gate Hospital, North Vancouver, British Columbia, Canada; 6 Division of Cardiology, Department of Medicine, Taipei Veterans General Hospital, Taipei, Taiwan; 7 Division of Infectious Diseases, Department of Medicine, Taipei Veterans General Hospital, Taipei, Taiwan; 8 Department of Family Medicine, Taipei Veterans General Hospital, Taipei, Taiwan

## Abstract

This study compared the risk of mortality in atrial fibrillation (AF) patients treated adherent to the 2012 European Society of Cardiology (ESC) guidelines for stroke prevention and those who were not treated according to guideline recommendations. This study used the Taiwan National Health Insurance Research Database. From 1996 to 2011, 354,649 newly diagnosed AF patients were identified as the study population. Among the study cohort, 45,595 and 309,054 patients were defined as Guideline-Adherent and Non-Adherent groups, respectively. During the follow up of 1,480,280 person-years, 133,552 (37.7%) patients experienced mortality. The risk of mortality was lower among AF patients whose treatment was adherent to the guideline recommendation for stroke prevention than those whose treatment was not (annual risk of mortality = 4.3% versus 10.0%) with an adjusted hazard ratio of 0.62 (95% confidence interval = 0.61–0.64, p value < 0.001) after adjusting for age, gender, CHA_2_DS_2_-VASc score and antiplatelet therapy. The findings were consistently observed after propensity matching analysis. In conclusion, the risk of mortality was lower for AF patients who were treated according to the antithrombotic recommendations of the 2012 ESC guidelines, guided by the CHA_2_DS_2_-VASc score. Better efforts to implement guidelines would lead to improved outcomes for patients with AF.

Atrial fibrillation (AF) is the most common and prevalent arrhythmia in the worldwide population^1^,^2^ and is independently associated with increased mortality and serious cardiovascular events such as ischemic stroke, transient ischemic attacks (TIA), and systemic thromboembolism^3^,^4^. Patients with AF have a five-fold increased stroke risk compared to patients in sinus rhythm^5^,^6^. Stroke patients with AF are characterized by a higher mortality, a prolonged hospitalization duration and a worse functional outcome^7^,^8^,^9^,^10^.

Oral anticoagulants (OACs) significantly reduce the risk of AF-related stroke^11^,^12^ but also increase the risk of bleeding^13^. The absolute benefit of OACs depends on the underlying risk of stroke. A simple assessment tool, the CHA_2_DS_2_-VASc (congestive heart failure, hypertension, age 75 years or older, diabetes mellitus, previous stroke or transient ischemic attack, vascular disease, age 65 to 74 years, female) risk score, is recommended and advocated in current guidelines to stratify individual stroke risk in patients with AF^14^,^15^. According to the European Society of Cardiology (ESC) guidelines, low-risk patients (defined by CHA_2_DS_2_-VASc score of 0 for males and 1 for females) do not require antithrombotic therapy. AF patients with ≥1 additional stroke risk factor beyond gender should be offered OACs for effective stroke prevention.

Despite the clear recommendations for stroke prevention in the guidelines, OACs are still under-used in real-world practice^16^,^17^,^18^, and adherence to treatment guidelines in AF patients is suboptimal^19^,^20^,^21^,^22^,^23^. A recent report from the EuroObservational Research Programme-Atrial Fibrillation (EORP-AF) Pilot General Registry, which enrolled 2,634 AF patients, demonstrated that ESC guideline-adherent antithrombotic management is associated with better outcomes^24^. However, the importance of guideline adherence for stroke prevention has not previously been well studied for Asian AF patients. The present study aimed to compare the risk of mortality in AF patients treated adherent or not adherent to the 2012 ESC guidelines for stroke prevention.

## Methods

### Database

The protocol of the present study is similar to our previous studies^25^,^26^,^27^,^28^,^29^,^30^. This retrospective study used the National Health Insurance Research Database (NHIRD) of the Taiwan National Health Research Institute. The National Health Insurance system is a mandatory universal program that offers comprehensive medical care coverage to all Taiwanese residents. Information in the NHIRD consists of detailed health care data from more than 23 million enrollees, representing >99% of Taiwan’s population. In this cohort dataset, the patients’ original identification numbers have been encrypted to protect privacy, with a consistent encrypting procedure to ensure linkage of claims belonging to the same patient and to allow patients to be followed over time.

Information about important co-morbid conditions of each individual was retrieved from the medical claims based on the International Classification of Diseases-Ninth Revision-Clinical Modification codes. We defined patients with a certain disease only when it was a discharge diagnosis or confirmed more than twice in an outpatient department. The diagnostic accuracies of important co-morbidities, such as hyperlipidemia, heart failure, hypertension, myocardial infarction, diabetes mellitus, and chronic obstructive pulmonary disease, have been validated previously^31^,^32^. Information about the medications the patients used was retrieved from the NHIRD using the specific code of each drug registered by physicians responsible for the treatment of the patients for the purpose of getting reimbursement from the National Health Insurance system. All anti-thrombotic drugs, including warfarin and aspirin, should be prescribed by physicians, and were not available over the counter in Taiwan.

### Study cohort

From January 1, 1996, to December 31, 2011, a total of 354,649 AF patients aged 20 years or older were identified from the NHIRD as the study population. AF was diagnosed using the International Classification of Diseases-Ninth Revision-Clinical Modification code (427.31) registered by the physicians responsible for treatment. To ensure diagnostic accuracy, we defined patients with AF when it was a discharge diagnosis or confirmed more than twice in the outpatient department^33^. The diagnostic accuracy of AF using the definition in the NHIRD was previously validated^34^,^35^.

The CHA_2_DS_2_-VASc risk score was calculated for each patient by assigning 1 point each for age between 65–74 years; history of hypertension, diabetes mellitus, recent heart failure, and/or vascular disease (myocardial infarction or peripheral artery disease); and female sex. Two points each were assigned for a history of stroke, TIA, or age 75 years or older^36^.

### Definition of guideline adherence

Guideline adherence was based on compliance with anti-thrombotic recommendations for AF stroke prevention according to the CHA_2_DS_2_-VASc score. According to the 2012 ESC AF guidelines, Guideline-adherent was defined as the following: (1) For AF males with a CHA_2_DS_2_-VASc score of 0 and AF females with a score of 1 (for gender), no anti-thrombotic drug was necessary, (2) For AF patients with an additional risk factor of the CHA_2_DS_2_-VASc scheme in addition to gender, an OAC should be prescribed. Patients who received treatment different from these recommendations were defined as “Non-adherent”. The clinical endpoint was defined as all-cause mortality.

### Propensity match analysis

We performed propensity score–matched analyses for patients adherent or non-adherent to ESC guideline. We calculated propensity scores for the likelihoods of adherence to guideline compared to non-adherence by multivariate logistic regression analyses, conditional on all baseline covariates listed in Table 1. After that, we matched patients who were adherent or non-adherent to ESC guideline with a 1:1 ratio on the basis of age, sex and the closest propensity score for the guideline adherence within a threshold of ±0.00001.

### Statistical analysis

Data were presented as the mean ± standard deviation (SD) for continuous variables and proportions for categorical variables. An unpaired 2-tailed Student *t* test was used for the analysis of continuous variables, and the differences between nominal variables were compared by the chi-square test. The incidence rate of all-cause mortality was calculated by dividing the number of events by person-time at risk with the 95% confidence interval (CI) estimated by using Fisher’s exact test. The risk of all-cause mortality was assessed using Cox regression analysis. Statistical significance was set at p < 0.05, and all statistical analyses were carried out with SPSS 17.0 (SPCC, Chicago, Illinois).

## Results

**Figure 1:**
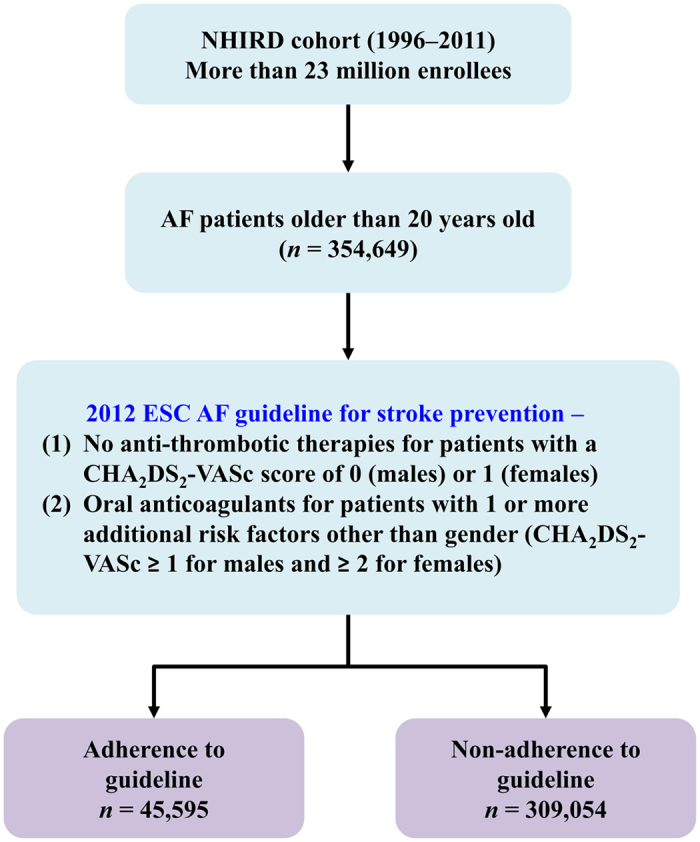
From 1996 to 2011, 354,649 AF patients older than 20 years old were identified from Taiwan’s NHIRD as the study population. Among the study population, 45,595 and 309,054 patients were defined as Guideline-adherent and Non-adherent groups, respectively. The risk of mortality was analyzed and compared between these two groups. **AF** = atrial fibrillation; CHA_2_DS_2_-VASc = congestive heart failure, hypertension, age 75 years or older, diabetes mellitus, previous stroke or transient ischemic attack, vascular disease, age 65 to 74 years, female; ESC = European Society of Cardiology; NHIRD = National Health Insurance Research Database.

**Figure 2:**
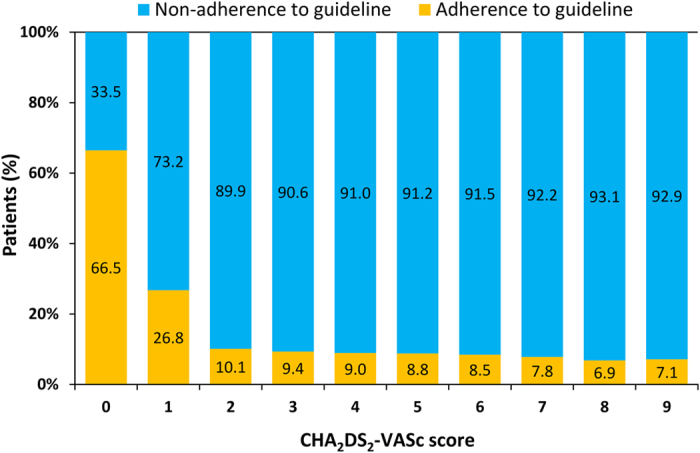
A large proportion of AF patients were managed non-adherent to the recommendations of the ESC guidelines on antithrombotic therapies for stroke prevention. In patients with a CHA_2_DS_2_VASc score of 2, only 10.1% patients were treated according to guidelines,with a trend towards lower guideline adherence among those at higher stroke risk (CHA_2_DS_2_VASc score of ≥3). CHA_2_DS_2_-VASc = congestive heart failure, hypertension, age 75 years or older, diabetes mellitus, previous stroke or transient ischemic attack, vascular disease, age 65 to 74 years, female; ESC = European Society of Cardiology; OAC = oral anticoagulant.

Figure 1 shows the flowchart of patient enrollment. Among 354,649 AF patients ≥ 20 years old, 45,595 and 309,054 patients belong to the Guideline-adherent and Non-adherent groups, respectively. The percentage of guideline adherence stratified by CHA_2_DS_2_-VASc score is shown in Fig. 2. The guideline-adherent rate was lower for patients with a high CHA_2_DS_2_-VASc score.

Table 1 shows the baseline characteristics of patients in each group. Guideline-adherent patients were younger, had fewer comorbidities, and had a lower CHA_2_DS_2_-VASc score compared to Non-adherent patients. A total of 43.8% of patients in the ESC guideline Non-adherent group received anti-platelet agents for stroke prevention.

**Figure 3:**
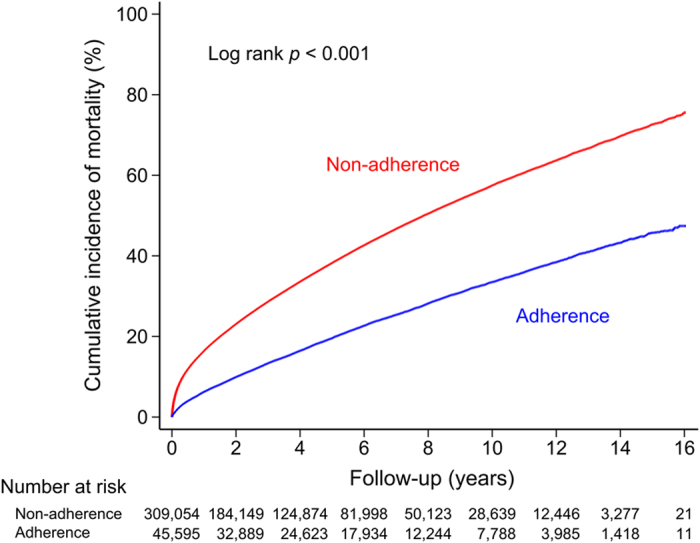
Log-rank test demonstrated a significantly different risk of all-cause mortality between the Guideline-adherent group and the Non-adherent group. As the cumulative incidence curve demonstrates, the Non-adherent group had a higher incidence rate of mortality than the Guideline-adherent group.

During the follow up of 1,480,280 person-years, the primary endpoint of mortality occurred in 133,552 (37.7%) patients. The risk of mortality was lower in Guideline-adherent AF patients than in Non-adherent AF patients (annual risk of mortality = 4.3% versus 10.0%), (Table 2). The cumulative mortality incidence curves of the two groups are shown in Fig. 3.

Table 3 shows the risk of mortality for patients who received treatment adherent to the ESC guidelines compared to those who did not. ESC guideline-adherent antithrombotic strategy was associated with a lower risk of mortality with an adjusted hazard ratio (HR) of 0.62 (95% CI = 0.61–0.64, p value < 0.001) after adjusting for age, gender, CHA_2_DS_2_-VASc score, baseline differences and the use of antiplatelet therapy (Table 3).

The baseline characteristics of patients in each group after propensity matching were shown in Supplemental Table 1. The propensity scores of 2 groups were similar. Age, sex and comorbidities were not significantly different between 2 groups. Similar to the results derived from the non-matched cohort, the annual risk of mortality was lower among patients who were adherent to guideline compared to the non-adherent group (4.9%/year versus 6.0%/year) with an adjusted HR of 0.80 (95% CI = 0.78–0.83, p value < 0.001) (Supplemental Tables 2 and 3).

## Discussion

To date, the present study is the largest retrospective analysis of mortality in AF patients based on whether their treatment was adherent or not adherent to the 2012 ESC guidelines for stroke prevention. The main finding of our study is that guideline-adherent antithrombotic management was associated with a 38% lower risk of mortality. Moreover, the overall guideline adherence rate was only 13% and even lower among patients with a high CHA_2_DS_2_-VASc score in this non-selected nationwide AF registry. These observations emphasize the importance of guideline adherence since it was associated with a better survival for AF patients.

The guideline adherence rate (13%) we observed in the present study is lower than in other observational studies, which cumulatively report an adherence rate between 53–75%^23^,^24^,^37^,^38^. One possible explanation for the differences is that previous guidelines used the CHADS_2_ score, rather than CHA_2_DS_2_-VASc, to stratify stroke risk, and treatment decisions were focused on identifying ‘high risk’ patients who should receive OACs. In contrast, the 2012 ESC AF guidelines adopted a more sensitive CHA_2_DS_2_-VASc score for stroke risk stratification and treatment focused on initial identification of ‘low risk’ patients who did not need antithrombotic therapy, and subsequently identified all others as patients for whom an OAC should be offered. Since more patients would be recommended to receive OACs when stratified by the CHA_2_DS_2_-VASc score as per the ESC 2012 guidelines, it may further widen the gap between guideline recommendations and the daily practices of the real world. Another plausible explanation could be that Asian patients are at a higher risk of warfarin-related intracranial hemorrhage (ICH) than other ethnicities^39^, and the concern of ICH may result in lower guideline adherence in Asia.

Several previous studies have investigated the impact of guideline adherence on the clinical outcomes of systemic thromboembolism (TE) and bleeding^23^,^37^. However, these previous studies focused on the 2001 and 2006 AF guidelines which adopted CHADS_2_ score for stroke risk stratification^23^,^37^, and data about the risk of mortality and adherence to CHA_2_DS_2_-VASc-based guidelines are limited. Recently, Lip *et al.* reported from the EORP-AF Pilot General Registry^24^ that event rates for ‘any TE’, the composite endpoints of ‘all-cause mortality & any TE’ and ‘cardiovascular death, any TE or bleeding’ were significantly lower in guideline-adherent patients. However, ‘any cause of death’ alone was non-significant (p = 0.0777), which may have been due to the relatively short follow-up duration and the small number of deaths. In the present study, which enrolled a large number of AF subjects followed for more than 1,480,000 person-years, the risk of mortality was 22% lower for patients whose treatment was adherent to the 2012 ESC AF guidelines for stroke prevention compared to those whose treatment was not. In the meta-analysis performed by Hart *et al.*, the use of warfarin not only reduced the risk of stroke by 64%, it also reduced the risk of all-cause mortality by about 25%^40^. Our study findings reinforce that guideline adherence can improve patient survival.

Older age and multiple comorbidities likely move physicians to discard OAC use precluding compliance with guideline recommendations. Similar to other observational studies^41^,^42^,^43^,^44^, our data showed that patients who were not treated according to guidelines were older, and have more co-morbidities and a higher CHA_2_DS_2_-VASc score. A common argument to withhold OAC in the elderly is the risk of major bleeding, particularly ICH. Certain patients with multiple comorbidities have a higher HAS-BLED (Hypertension, Abnormal Renal/Liver Function, Stroke, Bleeding History or Predisposition, Labile International Normalized Ratio, Elderly, Drugs/Alcohol) score, and an increased risk of major bleeding with OAC use^45^. However, the decision on use of OACs should be based on the stroke risk as assessed by the CHA_2_DS_2_-VASc score, reserving the HAS-BLED score to assist clinicians in identifying correctable risk factors for bleeding^14^. Supportively, a previous study demonstrated that patients were willing to endure 4.4 major bleeds in order to prevent one stroke^46^. Therefore, management of patients with a high risk for both ischemic stroke and bleeding should be based on joint and informed decision-making with patients, rather than based on the decision of physicians alone.

### Study limitations

This study was an observational study and has several limitations. First, patients were categorized into guideline-adherent or non-adherent groups according to the baseline antithrombotic treatments the patients received. The strategies for AF stroke prevention could be changed during the follow-up. We could not ascertain the drug adherence and the reason for non-adherence during the follow-up. However, at the timing when patients experienced mortality or at the end of follow up, more than 80% of patients were still categorized as the same groups as they were at the baseline. Furthermore, since patients who did not receive antithrombotic agents at enrollment were allowed to take OACs during follow-up according to the guideline recommendation and were not excluded from the analysis, the true risk of mortality for patients in the Non-adherent group could be even higher than what we reported here. Therefore, the true difference in mortality risk between these 2 groups could potentially be even larger. Second, the baseline characteristics were different between the 2 groups, and therefore we were not able to exclude the possibility that Non-adherent patients were older and sicker, which may have led to the observed increase in mortality. We adjusted for these potential confounders using multivariate Cox regression and propensity matching analyses. Third, we were not able to calculate the HAS-BLED (Hypertension, Abnormal Renal/Liver Function, Stroke, Bleeding History or Predisposition, Labile International Normalized Ratio, Elderly, Drugs/Alcohol) score for each patient because details on the amount of alcohol intake and biochemical indices of renal/liver function were not available in this registry database. The concern of severe bleeding for patients with a high HAS-BLED score may prohibit physicians from prescribing OACs, and a further study is necessary to explore the reasons why OACs were not used. Lastly, the present study only Taiwanese patients, and the suitability of result extrapolation to other populations remains uncertain.

## Conclusions

In this nationwide AF cohort, the risk of mortality was lower for patients who were treated according to antithrombotic recommendations of the 2012 ESC AF guidelines guided by the CHA_2_DS_2_-VASc score. Better efforts to implement guidelines would lead to improved outcomes for patients with AF.

## Additional Information

**How to cite this article**: Li, C.-H. *et al.* European Society of Cardiology Guideline-Adherent Antithrombotic Treatment and Risk of Mortality in Asian Patients with Atrial Fibrillation. *Sci. Rep.* **6**, 30734; doi: 10.1038/srep30734 (2016).

## Supplementary Material

Supplementary Information

## Figures and Tables

**Table 1:** Baseline characteristics of study patients.

Variables	Guideline-adherent (*n* = 45,595)	Non-adherent (*n* = 309,054)	*P* value
Age, years	61.0 ± 15.5	72.9 ± 11.9	<0.001
Age > 65, n (%)	19,665 (43.1)	244,176 (79.0)	<0.001
Age > 75, n (%)	9,972 (21.9)	155,066 (50.2)	<0.001
Male gender, n (%)	25,553 (56.0)	170,918 (55.3)	0.003
Comorbidities, n (%)			
Congestive heart failure	14,615 (32.1)	130,355 (42.2)	<0.001
Hypertension	20,607 (45.2)	222,215 (71.9)	<0.001
Diabetes mellitus	8,005 (17.6)	93,369 (30.2)	<0.001
Previous stroke/TIA	13,204 (29.0)	110,465 (35.7)	<0.001
Vascular disease	7,479 (16.4)	72,888 (23.6)	<0.001
Hyperlipidemia	9,114 (20.0)	79,478 (25.7)	<0.001
Chronic lung disease	10,101 (22.2)	115,792 (37.5)	<0.001
Liver cirrhosis	1,212 (2.7)	10,663 (3.5)	<0.001
ESRD	368 (0.8)	7,478 (2.4)	<0.001
Malignancy	2,013 (4.4)	17,527 (5.7)	<0.001
CHA_2_DS_2_-VASc score, median (IQR)	2 (1–5)	4 (3–6)	<0.001
Use of anti-platelet agents, n (%)	0 (0)	135,488 (43.8)	< 0.001

AF = atrial fibrillation; ESRD = end-stage renal disease; IQR = interquartile range; TIA = transient ischemic attack.

**Table 2:** Annual risk of mortality for AF patients whose treatment was adherent or non-adherent to the ESC guidelines for stroke prevention.

Groups	Number of events	Number of patients	Person-years	Incidence*
Guideline-adherent	10,512	45,595	244,415	4.3
Non-adherent	123,040	309,054	1,235,865	10.0

^*^Per 100 person-years of follow up.

**Table 3:** Hazard ratio for mortality in patients treated adherent or non-adherent to the ESC guidelines for stroke prevention.

Groups	Crude HR (95% CI)	*P* value	Adjusted^1^ HR (95% CI)	*P* value	Adjusted^2^ HR (95% CI)	*P* value
Non-adherent	reference		reference		reference	
Guideline-adherent	0.48 (0.47–0.49)	<0.001	0.76 (0.75–0.78)	<0.001	0.62 (0.61–0.64)	<0.001

HR, hazard ratio; CI, confidence interval, ESRD, end-stage renal disease.

^1^Adjusted for age, gender, and CHA_2_DS_2_-VASc score.

^2^Adjusted for age, gender, CHA_2_DS_2_-VASc score, hyperlipidemia, chronic lung disease, liver cirrhosis, ESRD, malignancy, and the use of anti-platelet agents.
