# Supplementary material for: European Society of Cardiology Guideline-Adherent Antithrombotic Treatment and Risk of Mortality in Asian Patients with Atrial Fibrillation
Source: Sci Rep. 2016 Aug 8;6:30734. doi: 10.1038/srep30734 (PMC4976390; doi:10.1038/srep30734)
Supplement: Supplementary Information [file srep30734-s1.doc]

**Supplemental Tables**

**European Society of Cardiology Guideline-Adherent Antithrombotic Treatment and Risk of Mortality in Asian Patients with Atrial Fibrillation**

Cheng-Hung Li, M.D.^1,2^*****, Chia-Jen Liu, M.D.^3,4^*****, Annie Y. Chou, M.D.^5^, Tze-Fan Chao, M.D.^2,6^, Ta-Chuan Tuan, M.D.^2,6^, Su-Jung Chen, M.D.^4,7^, Kang-Ling Wang, M.D.^2,6^, Yenn-Jiang Lin, M.D.^2,6^, Shih-Lin Chang, M.D.^2,6^, Li-Wei Lo, M.D.^2,6^, Yu-Feng Hu, M.D.^2,6^, Fa-Po Chung, M.D.^2,6^, Jo-Nan Liao, M.D.^2,6^, Tzeng-Ji Chen, M.D.^8^, Tsu-Juey Wu, M.D.^1,2^ and Shih-Ann Chen, M.D.^2,6^

^1^Division of Electrophysiology, Cardiovascular Center, Taichung Veterans General Hospital. ^2^Institute of Clinical Medicine, and Cardiovascular Research Center, National Yang-Ming University, Taipei, Taiwan. ^3^Division of Hematology and Oncology, Department of Medicine, Taipei Veterans General Hospital, Taipei, Taiwan. ^4^Institute of Public Health and School of Medicine, National Yang-Ming University, Taipei, Taiwan. ^5^Division of Cardiology, Department of Medicine, Lions Gate Hospital, North Vancouver, British Columbia, Canada. ^6^Division of Cardiology, Department of Medicine, Taipei Veterans General Hospital, Taipei, Taiwan. ^7^Division of Infectious Diseases, Department of Medicine, Taipei Veterans General Hospital, Taipei, Taiwan. ^8^Department of Family Medicine, Taipei Veterans General Hospital, Taipei, Taiwan.

***Dr. Cheng-Hung Li and Dr. Chia-Jen Liu contributed equally to this work.**

Conflict of interest: none declared.

Running title：ESC guideline adherence and risk of mortality in atrial fibrillation

Key words: atrial fibrillation, guideline adherence, antithrombotic management, mortality

Reprint requests and correspondence

**Tze-Fan Chao, M.D.**

Division of Cardiology, Department of Medicine, Taipei Veterans General Hospital,

No. 201 Sec. 2, Shih-Pai Road, Taipei, Taiwan.

Tel: 886-2-2875-7156, Fax: 886-2-2873-5656

E-mail: [eyckeyck@gmail.com](mailto:eyckeyck@gmail.com%0D%0C)

[**Supplemental**](mailto:eyckeyck@gmail.com%0D%0C) **Table 1. Baseline characteristics of study patients after the propensity match**

| Variables | Guideline-adherent  (*n* = 36,102) | Non-adherent  (*n* = 36,102) | *P* value |
| --- | --- | --- | --- |
| Age, years | 64.8 ± 13.5 | 64.8 ± 13.5 | 0.993 |
| Age > 65, n (%) | 19,271 (53.4) | 19,271 (53.4) | 1.000 |
| Age > 75, n (%) | 9,794 (27.1) | 9,794 (27.1) | 1.000 |
| Male gender, n (%) | 20,239 (56.1) | 20,239 (56.1) | 1.000 |
| Comorbidities, n (%) |  |  |  |
| Congestive heart failure | 13,752 (38.1) | 13,759 (38.1) | 0.957 |
| Hypertension | 20,030 (55.5) | 20,049 (55.5) | 0.887 |
| Diabetes mellitus | 7,567 (21.0) | 7,564 (21.0) | 0.978 |
| Previous stroke/TIA | 12,449 (34.5) | 12,452 (34.5) | 0.981 |
| Vascular disease | 6,929 (19.2) | 6,920 (19.2) | 0.932 |
| Hyperlipidemia | 8,062 (22.3) | 8,063 (22.3) | 0.993 |
| Chronic lung disease | 8,695 (24.1) | 8,697 (24.1) | 0.986 |
| Liver cirrhosis | 525 (1.5) | 519 (1.4) | 0.852 |
| ESRD | 181 (0.5) | 169 (0.5) | 0.520 |
| Malignancy | 1,116 (3.1) | 1,093 (3.0) | 0.619 |
| CHA_2_DS_2_-VASc score, median (IQR) | 3 (1–5) | 3 (1–5) | 0.990 |
| Propensity score | 0.20 ± 0.15 | 0.20 ± 0.15 | 1.000 |

AF = atrial fibrillation; ESRD = end-stage renal disease; IQR = interquartile range; TIA = transient ischemic attack

**Supplemental Table 2. Annual risk of mortality for AF patients whose treatment was adherent or non-adherent to the ESC guidelines for stroke prevention after the propensity match**

| Groups | Number of events | Number of patients | Person-years | Incidence* |
| --- | --- | --- | --- | --- |
| Guideline-adherent | 9,005 | 36,102 | 185,013 | 4.9 |
| Non-adherent | 10,885 | 36,102 | 181,330 | 6.0 |

* Per 100 person-years of follow up

**Supplemental Table 3. Hazard ratio for mortality in patients treated adherent or non-adherent to the ESC guidelines for stroke prevention after the propensity match**

| Groups | Crude HR  (95% CI) | *P* value | Adjusted^1^ HR  (95% CI) | *P* value | Adjusted^2^ HR  (95% CI) | *P* value |
| --- | --- | --- | --- | --- | --- | --- |
| Non-adherent | reference |  | reference |  | reference |  |
| Guideline-adherent | 0.81 (0.79–0.83) | < 0.001 | 0.81 (0.79–0.84) | < 0.001 | 0.80 (0.78–0.83) | < 0.001 |

^1^ Adjusted for the propensity score

^2^ Adjusted for age, gender and CHA_2_DS_2_-VASc score

HR, hazard ratio; CI, conﬁdence interval
